# Examining the barriers and recommendations for integrating more equitable insomnia treatment options in primary care

**DOI:** 10.3389/frsle.2023.1279903

**Published:** 2023-11-27

**Authors:** Ivan Vargas, Mara Egeler, Jamie Walker, Dulce Diaz Benitez

**Affiliations:** Department of Psychological Science, University of Arkansas, Fayetteville, AR, United States

**Keywords:** insomnia, primary care, cognitive behavioral therapy for insomnia (CBT-I), sleep, underserved population

## Abstract

Chronic insomnia is the most common sleep disorder, occurring in ~32 million people in the United States per annum. Acute insomnia is even more prevalent, affecting nearly half of adults at some point each year. The prevalence of insomnia among primary care patients is even higher. The problem, however, is that most primary care providers do not feel adequately knowledgeable or equipped to treat sleep-related concerns. Many providers have never heard of or have not been trained in cognitive behavioral therapy for insomnia or CBT-I (the first line treatment for insomnia). The focus of the current review is to summarize the factors contributing to why sleep health and insomnia treatment have been mostly neglected, identify how this has contributed to disparities in sleep health among certain groups, particularly racial and ethnic minorities and discuss considerations or potential areas of exploration that may improve access to behavioral sleep health interventions, particularly in primary care.

## Introduction

Most reports suggest that the prevalence of chronic insomnia (or insomnia disorder) is at least 10% (NIH, 2005; Aernout et al., 2021). This means that, on a global scale, over 700 million adults experience clinically significant insomnia symptoms at any given time. These numbers are even higher (27%−48%) when acute or sub-clinical insomnia are considered (Ohayon, 2002; Ellis et al., 2012; Perlis et al., 2020). The public health implications are even greater considering that insomnia is a significant risk factor for several psychological and physical disorders (Taylor et al., 2007; Mai and Buysse, 2008). The economic burden of insomnia is also high, with an estimated yearly cost of over $83 billion in the United States alone (based on per-person direct and in-direct costs) (Daley et al., 2009). Other research supports that some groups, particularly racial and ethnic minorities, are exposed to additional barriers that elevate their risk for developing chronic insomnia (Pigeon et al., 2011; Grandner et al., 2013, 2016; Kalmbach et al., 2016), and that these disparities and inequities in sleep have become greater since the COVID-19 pandemic (Cheng et al., 2021). Efforts to reduce the burden of insomnia (and minimize disparities in risk factors and access to care) are therefore critical for improving overall health and wellbeing.

Fortunately, there are several treatment options for insomnia, including cognitive behavioral therapy for insomnia (CBT-I). CBT-I is a behavioral intervention that is highly efficacious (Morin et al., 1994; Smith et al., 2002; Harvey and Tang, 2003; Irwin et al., 2006; Mitchell et al., 2012; Koffel et al., 2015; Seyffert et al., 2016) and has been named the first-line treatment for chronic insomnia (Schutte-Rodin et al., 2008; Qaseem et al., 2016; Riemann et al., 2017; Wilson et al., 2019). It primarily targets the mechanisms believed to perpetuate chronic insomnia (i.e., decreased homeostatic sleep drive and a maladaptive conditioning pattern with one's bed) (Bootzin, 1972). The treatment effects of CBT-I are durable (Morin et al., 1999, 2009) and have been tested in various patient populations (Garland et al., 2014; Cunningham and Shapiro, 2018; Waters et al., 2020; Selvanathan et al., 2021) and settings, including primary care (Davidson et al., 2019). All this said, it is still not widely used or available. This is especially true for racial and ethnic minorities who have been historically underserved in this way (Jhnson et al., 2019). While there are several considerations and challenges to the widespread availability and use of CBT-I, the two primary barriers are (1) significant lack of or misinformation about sleep and its interventions, especially among health providers (Grandner and Chakravorty, 2017; Koffel et al., 2018), and (2) that CBT-I is, outside of a few exceptions (Manber and Simpson, 2016), almost exclusively offered by behavioral sleep medicine therapists in specialty clinics (i.e., there is an overall lack of therapists) (Bootzin, 1972). One way to potentially meet the need for insomnia treatment and improve overall sleep health, particularly among underserved populations, is to make CBT-I more available by incorporating it into routine practice in primary care settings.

Insomnia among primary care patients is a prevalent issue, with upwards to 50% of primary care patients reporting at least some insomnia symptoms and nearly 20% reporting chronic insomnia symptoms (Shochat et al., 1999; Arroll et al., 2012; Maire et al., 2020). This has larger health implications considering that patients with insomnia are more likely to endorse greater functional impairment, disability, and health care utilization (Simon and Vonkorff, 1997). Fortunately, over half of primary care patients with sleep problems do report their symptoms to their general physician (Aikens and Rouse, 2005). In fact, primary care doctors are the most consulted provider when it comes to sleep-related issues. According to a study by Morin et al. (2006), of those patients that did consult a medical professional about their insomnia, 83% consulted their primary care physician. The concern, however, is that most general practitioners do not feel adequately equipped to treat or refer patients for insomnia treatment. One study, for example, surveyed a group of primary care providers within the Veterans Affairs (VA) Health Care System and found that the most commonly used treatment methods included sleep hygiene recommendations (i.e., a suboptimal treatment) or pharmacotherapy (Ulmer et al., 2017). This is consistent with prior reports suggesting that psychoactive medications were the most common treatment option for insomnia (Simon and Vonkorff, 1997; Falloon et al., 2011; Moloney et al., 2019). Part of the reason for this is that nearly 60% of providers had not heard of CBT-I or did not understand how it works. Nearly half of those providers also did not know where to refer patients for CBT-I or whether it was available in their clinic (Ulmer et al., 2017). The results from this study are surprising, however, considering that the VA is responsible for the only large-scale efforts to incorporate CBT-I into primary care, including a national roll-out of provider training for CBT-I (Manber and Simpson, 2016) and the development of web-based programs for augmenting CBT-I (i.e., CBT-I Coach, Insomnia Coach, and Path to Better Sleep) (Kuhn et al., 2016, 2022; Ulmer et al., 2018). This means that the percentages reported in this study are likely higher among non-VA providers. That said, it is still the case that even VA provider and patient knowledge related to the diagnosis and treatment of insomnia is limited (Bramoweth et al., 2020, 2021).

Considering the issues with the dissemination and implementation of insomnia treatment (and that minoritized individuals have been historically underserved) the current review aims to: (1) summarize the potential factors or barriers contributing to why sleep health and insomnia treatment have been mostly neglected, (2) identify how this has contributed to disparities in sleep health and insomnia treatment among racial and ethnic minorities, and (3) discuss future considerations or potential areas of exploration that may improve access to behavioral sleep health interventions, particularly as it relates to utilizing primary care services to increase access to CBT-I among underserved populations (i.e., racial and ethnic minorities). It is worth noting that the current review is on adult behavioral sleep medicine and adult-focused providers. While several of the issues presented here also apply to pediatric behavioral sleep medicine, reviewing this literature is beyond the scope of this paper. Similarly, the review focuses primarily on the assessment and treatment of insomnia. The integration of sleep medicine and the treatment of other sleep disorders into primary care has been previously reviewed elsewhere (Pagel and Pandi-Perumal, 2014).

## Reasons why sleep health and insomnia treatment have been neglected

Despite effective behavioral interventions for sleep and insomnia, the assessment and behavioral treatment of sleep remains elusive. This is likely because sleep interventions are (1) mostly unknown to both practitioners as well as the general population, (2) unavailable to those who do seek them out, (3) not a priority compared to other psychological and medical conditions, and (4) too burdensome and/or expensive for most individuals.

While CBT-I is the suggested treatment for chronic insomnia, many physicians still do not recommend it for their patients. One study that aimed to highlight the referral practices and attitudes toward insomnia treatments in the United States found that physicians saw an average of 15.2 patients with insomnia complaints per month. However, only 1.5 of those patients were referred for CBT-I. Only 9.2% of physicians believed that CBT-I alone was the most effective treatment approach (Conroy and Ebben, 2015). When different medical providers were asked about their insomnia treatment practices, practitioners were more likely to recommend pharmacotherapy, sleep hygiene, and relaxation therapy (Moss and Lachowski, 2013). Many still believed sleep hygiene education to be an effective stand-alone treatment despite little empirical support for its efficacy, and in some cases, evidence that it may lead to worse patient outcomes (Morgenthaler et al., 2006; Chung et al., 2018). Similar results were seen when surveying VA primary care providers. While 20%−39% of veterans reported insomnia symptoms, only 29% of providers referred clients to a provider trained in CBT-I (Ulmer et al., 2017).

One reason that practitioners may not be recommending CBT-I is simply because they lack familiarity with the intervention. When VA primary care providers were asked how familiar they were with CBT-I, 82.3% reported that they had heard of it, but only 9.8% felt like they had had good understanding of how it works (Ulmer et al., 2017). When general practitioners were asked about their treatment methods for insomnia many suggested that before they would consider using CBT-I they would want to see positive evidence that it is beneficial to patients (Davy et al., 2015). This suggests that practitioners had not been informed about the utility of CBT-I and may offer inaccurate information about the treatment (e.g., that CBT-I is equivalent to sleep hygiene education) (Koffel et al., 2020).

Understandably, if health care providers do not feel as though they have a good understanding of a treatment, it is not something they will recommend. This then leads one to ask, what education do providers receive regarding sleep assessment and treatment? A survey of medical schools found that, on average, only 2.5 h were spent on sleep education, with 27% of programs providing no education at all (Mindell et al., 2011). Along the same lines, residency programs spend an average of 4.7 h per year discussing sleep. While programs such as neurology, pulmonology, and critical care medicine retain 2–3 faculty members who specialize in sleep medicine, others like family medicine, psychiatry, and otolaryngology typically have none (Sullivan and Medicine, 2021). When sleep education is provided, it is usually in the form of lectures, journal clubs or grand rounds speakers, and not full-semester courses. The result is that physicians report limited knowledge about the assessment and treatment of sleep disorders (Owens, 2001; Khawaja et al., 2017).

While there is little to no sleep coverage in medical school education, the education of clinical psychologists is not much better. For example, in Australia, psychology graduate students reported receiving a median of 2 h of sleep education in their postgraduate programs, with 47% reporting no sleep education (Meaklim et al., 2021). In the United States and Canada, clinical psychologists reported a median of 10 h of sleep training across their whole career, with the vast majority reporting no sleep training during graduate school, internship, or post-doctoral fellowships (Zhou et al., 2020). This lack of training has also lowered psychologists' confidence in managing sleep disturbances in general and has left a considerable proportion of psychologists (40.5%) feeling not or only “a little” prepared to evaluate and treat insomnia.

This lack of sleep education has led to a short supply of behavioral sleep medicine specialists that can provide insomnia treatment to those who do actively seek it out. As of 2022, 205 individuals globally had been credentialed by the Board of Behavioral Sleep Medicine (BBSM). This has then led to most accredited sleep disorder centers not having full-time or part-time clinicians who specialize in the treatment of insomnia (Perlis and Smith, 2008). The few providers that are available are often clustered into a small number of states and cities. A 2015 survey found that 58% of behavioral sleep medicine providers resided in 12 states (CA, NY, PA, IL, MA, TX, FL, OH, MI, MN, WA, and CO), leaving states such as Hawaii, New Hampshire, South Dakota, and Wyoming with no providers. Providers also tend to be in larger cities, such as Chicago, New York, Philadelphia, San Francisco, and Los Angeles. However, other heavily populated cities in the United States, such as Jacksonville and San Jose, have no providers (Thomas et al., 2016).

In addition to sleep interventions being both unknown and unavailable, poor sleep is often not prioritized like other psychological and medical conditions. For example, a group of French-speaking Canadians were surveyed about their sleep, and when those with insomnia were asked how they tried to improve their sleep, less than half reported consulting a health care provider (only 13% of all respondents had ever consulted a physician about their sleep) (Morin et al., 2006). Instead, many reported using at home strategies such as herbal and dietary supplements, over the counter medications, relaxation strategies, and alcohol. When individuals sought consultation for their poor sleep, it was normally due to elevated levels of fatigue, psychological and physical discomfort, and reduced work productivity. While symptoms of insomnia can encourage some to talk with their primary care providers, others may not perceive them to be problematic enough to seek help. Individuals may believe that that their poor sleep will simply get better on its own or that it has been going on for so long that there is no need to pursue help. A qualitative analysis of patients' help-seeking behaviors suggested that insomnia and other sleep problems are typically only reported when patients are seeking help for a different health ailment (Cheung et al., 2014). Another study found that people were more likely to talk with their primary care physicians about their sleep if they already had multiple different medical conditions and believed that their sleep problem affected their daytime functioning (Aikens and Rouse, 2005).

Individuals who are interested in treating their poor sleep, are educated about behavioral interventions, and have a provider in their area may still be unable access treatment as it may be too expensive. While there is no set cost of CBT-I, individuals may expect to spend anywhere between $200 and $2,500 depending on the clinician, location, and number of sessions. While many insurances will cover cognitive behavioral therapy, they do not all uniformly reimburse behavioral sleep medicine services (Perlis and Smith, 2008). Access is even more limited for those who live in rural settings as insurances like Medicare will only reimburse a limited number of telehealth sessions. It can also be costly for sleep clinics as insomnia services are typically billed as mental health services and many sleep disorders centers do not have the credentials or administrative infrastructure to provide behavioral therapy for insomnia.

If an individual does get past all these barriers and begins CBT-I, they may find that the treatment itself can be quite burdensome. A standard course of CBT-I can typically take anywhere from 6 to 12 weeks (about 3 months). This timeline can extend even further if sessions are delivered bi-weekly. CBT-I treatment studies, for instance, often see high dropout rates with most individuals stopping between the 4th and 5th session (Ong et al., 2008). When individuals are already less willing to prioritize their sleep, it may be more difficult for them to dedicate this extensive amount of time to treatment. As a result, many primary care providers typically do not prescribe nonpharmacological treatments for sleep due to lack of interest and motivation from the patients (Dollman et al., 2003; Anthierens et al., 2010). To make matters worse, most primary care providers are not aware that CBT-I can be adapted or abbreviated (Nowakowski et al., 2022). While a standard course of CBT-I is the primary clinical recommendation for patients with chronic insomnia or insomnia disorder, patients with acute or sub-clinical insomnia can still benefit from an abbreviated course of treatment (e.g., four sessions or even one session) (Ellis et al., 2015; Boullin et al., 2017; Kwon et al., 2021).

## Disparities in sleep and insomnia among racially and ethnically diverse individuals

Although race and ethnicity are socially constructed concepts used to classify groups of people, they are useful to the extent that they provide a “lens through which to study disparities and inequities in health and health care” (Flanagin et al., 2021). Importantly, racial and ethnic disparities in sleep occur due to a variety of reasons, including socioeconomic disadvantages, racism, immigrant stress, neighborhood characteristics, health conditions, discrimination and other intergenerational systems that impede good sleep. According to Williams et al. (2015), it is important to identify and address the barriers that prevent Black, Indigenous, and other People of Color (BIPOC) communities from getting good sleep. The goal of this review is to *introduce* the reader to some of these barriers for BIPOC individuals and how increased access to behavioral sleep medicine in primary care may help address some of these disparities and inequities. While there are a number of social and environmental factors (e.g., SES or community distress) that overlap with or explain racial and ethnic disparities in sleep, we do not exhaustively review the mechanisms by which each one of these factors contributes to disparities. The focus here is on broadly highlighting the systemic barriers that affect the pursuit of good sleep health and minimize the negative effects of living with insomnia, especially among BIPOC communities, and providing suggestions for how the field may address these barriers using a primary care model of sleep health. Future efforts to unpack the specific role of each of these social and environmental factors are important and needed and should be the focus of future work.

Most racial and ethnic disparities research studies in sleep health have been conducted using Black and White participants. A systemic review examining 97 studies related to sleep disparities and race found that 80% of studies compared White and Black participants, while only 40% included Hispanic participants, 38% included Asian participants, and only seven studies included Native American and Pacific Islanders (Ahn et al., 2021). Additionally, when Hispanic and Asian participants were included, their sample size was much smaller compared to other races and ethnicities. This review also suggested that racial and ethnic minorities in the United States got less sleep compared to non-Hispanic White participants and that Black participants had lower sleep efficiency than White participants consistently across many studies. Similarly, results from another study found that non-White participants, which included Black, Asian, and Hispanic participants, reported worse sleep quality and duration and more daytime sleepiness than non-Hispanic White participants (Carnethon et al., 2016). Black women in this study averaged 5.9 h of sleep per night and Black men averaged 5.1 h per night, whereas White women in this study averaged 6.7 h. Lower sleep duration for Black, Hispanic, and Asian participants compared to White participants was present even when controlling for other factors such as education, work schedule, BMI, and other health variables. When comparing across nativity status, Black immigrant workers were found to have shorter sleep than U.S. born Black workers (Jackson et al., 2014). Black workers, whether immigrant or U.S. born, still reported the least amount of sleep out of all racial and ethnic groups and across all job types. According to meta-analytic norms, Black individuals have worse sleep on both subjective and objective metrics of sleep continuity and duration when compared to White individuals (Ruiter et al., 2011). The results from another systematic review found that, compared to non-Hispanic white adults, Asian, Black, Hispanic and Latine adults reported lower sleep duration and quality. Alternatively, little or insufficient evidence is available for adults who identify as American Indian, Alaska Native, Native Hawaiian or Pacific Islander (Jhnson et al., 2019).

In general, racial and ethnic differences in insomnia has been understudied and the literature that is available has provided mixed results. That said, several studies do now support that BIPOC populations, especially those individuals who identify as Black or African American, are at greater risk for experiencing insomnia symptoms and insomnia disorder (Ruiter et al., 2010; Grandner et al., 2013; Chen et al., 2015; Kalmbach et al., 2016; Cheng et al., 2021). For example, the Multi-Ethnic Study of Atherosclerosis (MESA) assessed insomnia rates among a large sample of racially and ethnically diverse adults (age 54 years and older) and found that Black adults reported higher rates of insomnia compared to White adults (Chen et al., 2015). Support for racial and ethnic differences in insomnia among other groups is less clear, with some findings suggesting that individuals who immigrate to the United States (i.e., not U.S. born) report lower rates of self-reported insomnia (Seicean et al., 2011; Hale et al., 2014). One potential explanation for this may be related to underreporting among BIPOC or immigrant populations. For example, one study asked participants about their sleep complaints: “Over the last 2 weeks, how often have you had trouble falling asleep or staying asleep or sleeping too much?” (Grandner et al., 2010). Participants indicated how many nights they had sleep complaints. Overall, non-White participants had less sleep complaints than White participants. Similarly, Phillips and Mannino (2005) found that Black participants had less sleep complaints than White participants. While underreporting may be due to repressive coping of life stresses (Jean-Louis et al., 2007), lack of knowledge about the importance of sleep (Gradisar et al., 2013), or a distrust in medical and research professionals due to historical racism and abuse in medical and research settings, it does create an important barrier to implementing strategies that promote better sleep or insomnia treatment among BIPOC individuals. Future research is needed to better understand the specific reasons that BIPOC individuals minimize or underreport sleep-related and insomnia complaints.

These disparities occur in overall sleep patterns and in treatment for sleep disturbance. BIPOC populations face patient-level, provider-level, and healthcare system level barriers to treatment. At the patient level, lack of comfort with the English language may hinder someone from seeking services (Fiscella et al., 2022). Additionally, help seeking attitudes or stigma may hold people back from seeking care for sleep difficulties. Treatment adherence in these communities also tends to be lower when compared to White patients. For example, patient engagement and treatment adherence has been found to be lower in Black women being treated with CBT-I (Kalmbach et al., 2023). Black communities are therefore less likely to be diagnosed and follow through with treatment. There have been recent efforts, however, to culturally-tailor CBT-I for black women and these efforts have been successful in more effectively engaging participants in the process without compromising the overall effectiveness of the intervention (Zhou et al., 2022). Provider-level barriers could include provider bias and lack of cultural competence training. For example, Williams et al. (2015) suggested that Black patients may be more likely to be prescribed medication rather than be referred out to a specialist due to provider biases about treatment adherence. System level barriers also limit access to care. For example, BIPOC populations are more likely to not have insurance or be under-insured. Even when they are insured, there are not enough sleep experts to treat them, or insurance may not cover the cost of these types of services. Some research studies have attempted to tackle these disparities in sleep by using culturally informed practices for recruitment, participation, and retention in studies. For example, studies have strategically recruited from places where they can reach more BIPOC participants, such as churches, community centers, or barber shops (Seixas et al., 2018). This study found that involving a peer health educator that lives in the same community made it more likely that participants followed through on scheduling follow-up appointments.

In conclusion, providers have a responsibility to help minimize sleep health disparities for BIPOC populations. These communities require interventions that target the problem and work at the individual, provider, and systemic levels. While this review focuses on disparities related to race and ethnicity, it is the case that other social and cultural factors play a key role in dictating sleep health behaviors, such as socioeconomic status (or SES), employment and work schedules, neighborhood safety and other characteristics, caretaking responsibilities, and discrimination/racism (Basner et al., 2007; Grandner et al., 2010; Jackson et al., 2014; Gamaldo et al., 2015; Jehan et al., 2018). Additional work in this area is needed to develop strategies for how to close the gap and limit disparities in sleep and insomnia.

## Considerations for incorporating sleep interventions (e.g., CBT-I) into primary care settings to increase overall access for underserved populations

### Increased sleep health education

One of the first steps necessary to incorporate sleep interventions into primary care settings is to increase public health education about the importance of sleep. General public sleep health education is an underrecognized opportunity to impact a wide range of health outcomes such as cardiovascular disease, obesity, mental health, and neurodegenerative disease (Hale et al., 2020). Topics could focus on the importance of sleep (e.g., what is good sleep, what is enough sleep, what are the consequences of not getting enough sleep or quality sleep). And, in the case of primary care patients, more education on the impact and relevance of including sleep as part of their treatment plan would provide a more individualized opportunity for education. Furthermore, sleep problems can play a causal role in the development of other conditions. This knowledge may serve as a motivating factor not only for primary care providers to prioritize sleep, but their patients to prioritize it as well. Public health campaigns, especially those targeted to underserved populations (i.e., racial and ethnic minorities), may facilitate greater public knowledge about the importance of sleep health. Research efforts to determine the factors (e.g., what language is used, what format is used to deliver the information, who is delivering the information) that will influence the effectiveness of these campaigns are needed.

Patient education should also include efforts to inform patients and the general public about the effectiveness of CBT-I (Koffel et al., 2018). Although sleep aids are routinely requested and prescribed, both patients and physicians have consistently expressed negative attitudes toward the use of sedative hypnotics (Moloney, 2017). One route of disseminating education to patients about the benefits of behavioral sleep interventions could be educational brochures about the harms and hazards of hypnotics (with CBT-I as a safer alternative) provided in waiting rooms of primary care settings (Silverstein et al., 2016). Educating patients about insomnia and CBT-I could be helpful in providing a more balanced view of treatment options. While individual-level behavioral sleep recommendations are important, sleep health promotion interventions could make even more of an impact if they occurred at multiple contextual levels (e.g., family, schools, workplaces, media, and policy) (Hale et al., 2020).

### Increase basic sleep health training

Another step required for the implementation of sleep interventions into primary care settings is to increase sleep health training at all levels. One suggestion is to broaden the provider base to include master's level practitioners so that clinicians other than clinical psychologists can offer CBT-I as well. For example, nurse practitioners and physician's associates are well-positioned because of their training in medical assessments, taking a biopsychosocial approach to conceptualization, and primary care skills. Furthermore, their ability to bill under a medical rather than mental health coverage code means that they could be easily integrated into various medical settings (Perlis and Smith, 2008). One study found that primary care nurse practitioners (who received training and ongoing supervision in CBT-I) treated patients who subsequently experienced initial and sustained improvements in sleep quality after a course of CBT-I treatment (Espie et al., 2007). Incorporating CBT-I training into nursing school curricula would not only increase access in underserved areas and communities but would also increase visibility and awareness of behavioral treatments for insomnia (Fields et al., 2013).

Many studies have shown that master's level practitioners provide care leading to outcomes that meet or exceed standard outcomes. Research has also shown that clinicians can provide CBT-I successfully without being a certified provider of comprehensive behavioral sleep medicine or having a doctoral degree (Fields et al., 2013). Social workers and social work students are a good example of a master's level provider that help fill this gap, especially for underserved populations that are more likely to have access to social work services than specialized behavioral sleep medicine services. Like other professional programs, social work programs include little to know training in sleep health or insomnia, despite student desire to learn more about sleep and overall relevance to the work that socials worker do (Spadola et al., 2023). Overall, the utilization of master's level clinicians would make access to insomnia treatment more affordable and accessible.

Additionally, the importance of sleep across the practice of medicine should be prioritized with consistent offerings of full courses, modules, didactics, practicums, and clinicals. In other words, more people should be able to deliver CBT-I-like interventions. Meaklim et al. (2023) recently provided an example of how this type of training could be disseminated more broadly. Ideally, all providers would either know how to deliver behavioral sleep interventions, where to refer people, or what resources to provide them. This process would not only include training in how to effectively use a referral stream, but also how to develop one, requiring a networking component that advertises various sleep health services in one's community. Utilizing a primary care liaison to facilitate referrals for outside of primary care could also be helpful. This primary care liaison model has shown to foster ongoing interactions between primary care and other agencies, connect patients to relevant programs/services, and could optimize the referral process to sleep providers (Boll et al., 2021).

Research on the experiences and perceptions of clinicians regarding the primary care management of insomnia has revealed that clinicians would like to have improved education about treatment options for insomnia (Davy et al., 2015). These educational interventions could address gaps in clinician knowledge in areas such as etiological models of insomnia, adverse health outcomes associated with insomnia, and effectiveness and application of CBT-I (Koffel et al., 2018). Increased training in behavioral sleep medicine could also lead to clinicians feeling more comfortable with the assessment and treatment of insomnia. There are several diagnostic tools and behavioral interventions that can be integrated into primary care. More training will allow providers to feel equipped to give recommendations beyond sleep hygiene education (Irish et al., 2015).

### Increase advanced sleep health training

Another strategy is to increase advanced sleep training at all levels, with the goal of increasing the number of sleep “experts” across all health disciplines. In other words, more people should be able to deliver CBT-I in complex cases of chronic or comorbid insomnia. Education in various stages of training and throughout a provider's career is important for facilitating the dissemination and implementation of behavioral sleep interventions (Vargas and Perlis, 2020). Education about behavioral interventions as a first line treatment (or addition to medication) should reach all types of primary care providers (RN's, NP's, APRN's, PA's, MD's, DO's, etc.), as well as PhD's and other clinicians providing psychotherapy services. Increased access to advanced training for providers would include behavioral interventions for sleep and would focus on a target audience beyond psychologists. This training would likely increase comfort level with giving direct advice and instructions about how to improve sleep. These efforts may also help move CBT-I beyond the BSM “hotspots” and make CBT-I more accessible in areas with little to no BSM specialists. One way this could be achieved is by incentivizing the delivery of care from DBSM's in underserved states or regions.

### Adapt behavioral sleep interventions

Another way to increase overall access to sleep health treatment is to prioritize efforts to adapt and reimagine what sleep interventions look like. There are at least two general considerations when adapting behavioral interventions, such as CBT-I. The first is related to adaptations specific to being able utilize CBT-I in a primary care setting and the second is related to cultural adaptations.

There are several ways to incorporate CBT-I into primary care in a way that makes sense. For example, the need for CBT-I treatment outweighs its availability as an individually delivered therapy, so group treatment could be an attractive alternative. Another approach is one session “inoculation.” One study found that a single session of CBT-I is an effective treatment for patients with acute insomnia when integrated into the stepped care model of insomnia (Ellis et al., 2015). Another option for brief insomnia treatment is Brief Behavioral Treatment for Insomnia (BBTI). In a study implementing two intervention sessions and two telephone calls delivered by a nurse clinician, treatment outcomes (self-reported sleep and health, sleep diary, actigraphy) were significantly better in the BBTI group than in the control group, and improvements were maintained at 6 months (Buysse et al., 2011). These results indicate that BBTI is a simple, effective, and lasting intervention for chronic insomnia. Another option would be a hybrid approach to CBT-I, utilizing two intervention sessions followed by 2 months of self-help treatment. Some research also suggests that incorporating stepped-care models into the implementation of CBT-I would increase the likelihood that appropriate levels of care could be delivered in different settings. For example, mild to moderate insomnia could be addressed in a primary care setting, while severe or comorbid insomnia could be referred to a specialty sleep clinic or advanced-level provider (Espie, 2009). A stepped care model would also allow master's level providers to manage less complex or acute presentations of insomnia, while triaging more complex or chronic cases and those with comorbidities to be referred to behavioral sleep medicine specialists (Neylan, 2011; Vincent and Walsh, 2013). This approach to behavioral sleep medicine could increase patient access to CBT-I providers and professionals with expertise in behavioral sleep medicine (Koffel et al., 2018).

Cultural adaptations are another priority that may help with the acceptability and implementation of CBT-I. To date, several studies have made adaptations to CBT-I that focus on the needs of specific underserved populations (e.g., veterans, women with breast cancer) (Alcántara et al., 2021). Very few studies, however, have investigated how to culturally tailor behavioral sleep interventions for individuals who are racially or ethnically diverse. One of the few studies to do this modified an internet-based version of CBT-I (i.e., SHUTi) for black women (Zhou et al., 2022). They used, for example, photos and videos of Black actors to present the information in the program. In addition, the investigators modified the didactic content based on the recommendations from relevant stakeholders they interviewed (i.e., black women). These recommendations included making changes to the social and cultural context of the information that was provided (e.g., implementing stimulus control in a crowded home environment). Another study that interviewed racially and ethnically diverse adults also found that adaptations to behavioral sleep interventions should consider barriers related to interpersonal, organizational and environmental factors (Rottapel et al., 2020).

## General considerations and future directions

There is room for improvement in the treatment of insomnia in primary care, specifically in the implementation of CBT-I. Increasing the availability of CBT-I in primary care is important because it can help relieve the burden and functional impairment caused by insomnia, especially among groups that have been historically underserved (i.e., racial and ethnic minorities). In addition, the treatment of insomnia in primary care can function as a form of primary and secondary prevention for other mental and physical health disorders, given that insomnia is a known risk factor for a number of conditions, such as depression and suicidality (Kwon et al., 2021; Nowakowski et al., 2022). Because of the practical limitations associated with delivering clinical services in a primary care setting, abbreviated or single-session versions of CBT-I may prove to be useful, especially for patients with acute (as opposed to chronic) insomnia. Primary care and integrated behavioral health care (IBHC) settings are an ideal opportunity to offer just-in-time intervention for patients suffering from sub-clinical insomnia. In a primary care or IBHC model, patients are referred to an in-clinic BHC for brief behavioral interventions, with appointments typically lasting 15–30 min. These interventions typically include only a limited number of follow ups that focus on improving patient day-to-day functioning related to a wide variety of mental and physical health concerns. Treating insomnia in this setting is both possible and necessary. In conclusion, literature on how traditional CBT-I compares to alternative delivery methods and alternative practitioners remains elusive. There are steps we can take as a field. In Table 1, we summarize some of the considerations and recommendations that were offered in this paper. Moreover, we can also continue to utilize recommendations from other on how to, for example, use implementation science to evaluate different strategies for integrating CBT-I (or forms of CBT-I) into primary care settings (Parthasarathy et al., 2016; Bramoweth et al., 2018; Germain et al., 2021). Dissemination and implementation science will allow us to further study adapted versions of CBT-I and specifically test the effectiveness and scalability of these alternative formats. This research will guide future decision-making on best practices for incorporating CBT-I into primary care.

**Table 1:** Summary of key recommendations for future research and practice.

**1. Increase efforts to raise awareness and knowledge about sleep health and insomnia in the general public. This can begin with investigating what strategies are most effective in disseminating that knowledge (e.g., increased sleep health education in schools, sleep wellness check-ups from primary care doctors and pediatricians, or social media campaigns) and what factors are important when tailoring that information to certain communities (e.g., making sure the information is relatable and from a relevant source). This information should include the importance of sleep on overall health and well-being and what treatment options are available and effective (e.g., CBT-I) and for whom**
2. **Increase efforts to promote basic sleep and insomnia training among clinicians and healthcare professionals**. These efforts should be broadly aimed at increasing the behavioral sleep medicine workforce. This includes expanding the scope of who can provide psychoeducation about sleep and treatment for insomnia (even if in a limited way) to master's level providers, such as social workers, and primary care providers, such as physicians and nurses. Increased training efforts are needed at all levels, from incorporating sleep and insomnia education into standard training programs to national roll-outs or continuing education programs (both at the basic and advanced levels). The latter will likely need to include efforts to understand what will incentivize healthcare systems and providers to acquire this additional training
3. **Adapt behavioral sleep interventions to be more suitable with other healthcare systems or communities**. Traditional behavioral sleep interventions, such as CBT-I, are not compatible (e.g., in terms of number of sessions or session length) with the way that healthcare is delivered or reimbursed in the United States. More research is needed to develop and test behavioral interventions (i.e., modified versions of CBT-I) that can be easily integrated into routine practice. Some of these alternatives already exist (e.g., BBTI, “one-shot” CBT-I, mobile CBT-I) and therefore the next steps may be to design strategies for how to implement them into practice. Additional work is also needed to identify if and how CBT-I can be culturally-tailored to underserved populations. These efforts can target strategies for how to make CBT-I more effective and/or how to increase patient engagement with CBT-I

## Author contributions

IV: Conceptualization, Writing—original draft, Writing—review & editing. ME: Conceptualization, Writing—original draft. JW: Conceptualization, Writing—original draft. DB: Conceptualization, Writing—original draft.
